# Reduced ownership over a virtual body modulates dishonesty

**DOI:** 10.1016/j.isci.2022.104320

**Published:** 2022-04-27

**Authors:** Marina Scattolin, Maria Serena Panasiti, Riccardo Villa, Salvatore Maria Aglioti

**Affiliations:** 1 Sapienza University of Rome and CLN^2^S@Sapienza, Italian Institute of Technology, Rome (RM) 00161, Italy; 2 Santa Lucia Foundation, IRCCS, Rome (RM) 00179, Italy; 3 Department of Psychology, Sapienza University of Rome, Rome (RM) 00185, Italy

**Keywords:** Behavioral neuroscience, Machine perception, Applied sensory psychophysics

## Abstract

Although studies suggest that even higher-order functions can be embodied, whether body awareness may bias moral decisions toward (dis)honesty remains underinvestigated. Here, we tested if the Sense of body Ownership (SoO) and the magnitude of monetary rewards influence the tendency to act immorally. Through a virtual body, participants played a card game in which they could lie to others to steal high or low amounts of money. To manipulate SoO, the virtual body was seen and controlled from a first-person perspective, with hands attached or detached, or from a third-person perspective. In third-person perspective, SoO was significantly reduced and more egoistic lies were produced in high reward conditions. Thus, SoO reduction and high monetary reward facilitate dishonest behavior, likely by separating the self from the dishonest actions performed through the disowned body. Because most future interactions will likely occur in a digital metaverse, our results may have crucial societal impact.

## Highlights

• We modulated the sense of ownership over a virtual body while preserving agency
• Ownership is reduced when observing the virtual body from an outside perspective
• Low ownership increases lying to others if the largest monetary reward is at stake
• Body discontinuity decreases lies that give the largest reward to others

## Graphical abstract

**Figure:**
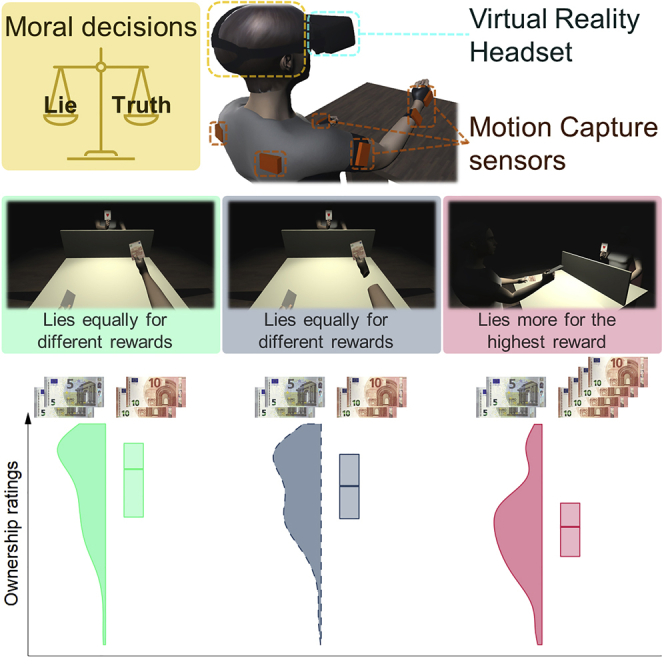

## Introduction

The variety of sensorimotor signals that bidirectionally connect the body and the brain are fundamentally important for the emergence of corporeal awareness, the complex mental construct mainly based on the feeling of having a body (sense of body ownership, SoO) and of controlling one’s actions and their consequences in the external environment (sense of agency, SoA) (Blanke, 2012; Haggard, 2017).

The SoO regards the body as a whole as well as its parts, which are all perceived as belonging to the self (Giummarra et al., 2008). Because of its reliance on multisensory signals, body ownership is not a stable percept but rather a ductile one: as information coming from sensory modalities is updated, so is the SoO. A variety of studies demonstrated that synchronous and spatially congruent stimulation modulates the SoO. At any given time, spatiotemporal congruence leads the brain to reorganize the incoming information into a new, coherent percept of the body (Ehrsson, 2020). For instance, when people feel tactile stimuli on their own body but simultaneously see them over a rubber limb (Botvinick and Cohen, 1998; Crea et al., 2015; Lenggenhager et al., 2015), another person’s face (Bufalari et al., 2014; Porciello et al., 2018; Sforza et al., 2010), or a full, virtual body (Lenggenhager et al., 2007; Slater et al., 2009), they feel that the observed limb, face, or body is their own. Similarly, the illusion of being looking at their own real body from behind is experienced if strokes are delivered to the chest and observed below one’s viewpoint, where the torso would be (Ehrsson, 2007). Crucially, information coming from senses other than touch also appears to contribute to the SoO. In fact, auditory and olfactory cues were found to strengthen the illusion of ownership over external objects (Radziun and Ehrsson, 2018; Roel Lesur et al., 2020) and even change the representation of one’s real limbs (Senna et al., 2014; Tajadura-Jiménez et al., 2015) and body (Clausen et al., 2021). However, the SoO does not exclusively rely on multisensory integration but also on the human-like appearance of the body and the perspective from which this is observed (Maselli and Slater, 2013; Monti et al., 2020). Indeed, unrealistic bodies and points of observation have been associated with significant reductions of the SoO towards virtual avatars. Moreover, studies found that observing a virtual hand that appears detached from a virtual forearm significantly reduced ownership ratings towards the separated, virtual hand in comparison with a connected one (Fusco et al., 2021; Tieri et al., 2015a, 2015b). Interestingly, the same reduction of SoO was observed for visually disconnected hands even when a synchronous, visuo-tactile stimulation was present (Perez-Marcos et al., 2012). In addition, participants report a lower SoO when they observe a virtual body from a third vs first-person perspective (Monti et al., 2020; Slater et al., 2010). Perspective over the body also contributes to the feeling of being located where the body is, or sense of location, and a reduction of the latter is oftentimes associated with diminished SoO (Serino et al., 2013). Although converging evidence suggests that modulations of the SoO are possible, the impact that conditions of heightened or reduced SoO have over higher-order mental functions is yet to be fully uncovered.

According to the embodied cognition perspective, bodily states play a central role in cognition (Barsalou, 2008). In keeping, feeling ownership toward bodies with different characteristics may induce changes in a variety of psychological functions. For instance, feeling that a childlike or a disproportionately tall or short body is one’s own can affect estimation of objects size as if one were a child or a very tall/short person (Banakou et al., 2013; van der Hoort et al., 2011). Similarly, the kinematics of the body changes in accordance with specific characteristics of the owned body. In fact, slowed gait was observed following embodiment of elderly avatars (Reinhard et al., 2020), and the velocity of reaching movements is reduced when action sounds create the illusion that one’s arm is elongated (Tajadura-Jiménez et al., 2016).

Notably, even higher-level cognitive functions, such as fluid intelligence or problem-solving, appear to be affected by the characteristics of the body we feel ownership toward (e.g., an ape-like or Einstein-like avatar; Banakou et al., 2018; Ma et al., 2019). A similar impact of the owned body has been observed in social cognition. For example, embodiment of a virtual superhero prompts more prosocial behaviors than identification with a virtual villain (Yoon and Vargas, 2014). In addition, having a SoO over bodies that differ from the real one in terms of skin color can reduce implicit biases against individuals of the same race (Maister et al., 2015; Peck et al., 2013) and increase the tendency to mimic their actions (Hasler et al., 2017). Similarly, when male participants experience SoO over a female avatar, implicit prejudice against women appears to reduce (Gonzalez-Liencres et al., 2020), whereas the erogeneity of vicarious touch delivered by male avatars increases (Mello et al., 2021). Hence, it seems plausible that our interactions with others are affected by fluctuations in the SoO.

Yet, investigations focusing on how the SoO over a virtual body can affect moral behaviors are scant. Some studies suggest that dishonesty may decrease in conditions where people experience low levels of body ownership, with less awareness of one’s own bodily needs and desires reducing the value associated with rewards (Paulus, 2007a; 2007b). Accordingly, previous investigations suggest an association between awareness of one’s bodily signals and an increase of egoistic, self-serving behaviors (Lenggenhager et al., 2013; Mancini et al., 2011). However, it has been shown that generosity increases with the degree of sensitivity toward information coming from the body (Piech et al., 2017). In line with this, a recent study found that the SoO over one’s own physical body is positively associated with how importantly individuals value having certain moral characteristics (Scattolin et al., 2021b). Interestingly, these results suggest that the opposite relation between SoO and morality may apply, with conditions of heightened SoO leading individuals to behave less dishonestly. Such relation may find its explanation in the hypothesis that an enhancement of the SoO facilitates the attribution of characteristics associated with the owned body to the self (Lee and Schwarz, 2021; Scattolin et al., 2021a). Conversely, a reduction of the SoO could create a sense of separation from the body and therefore protect one’s image of self from being associated with its negative aspects. Thus, lowered SoO may also represent a way to separate the self from the consequences of immoral behaviors, which then could become more likely to occur.

**Figure 1:**
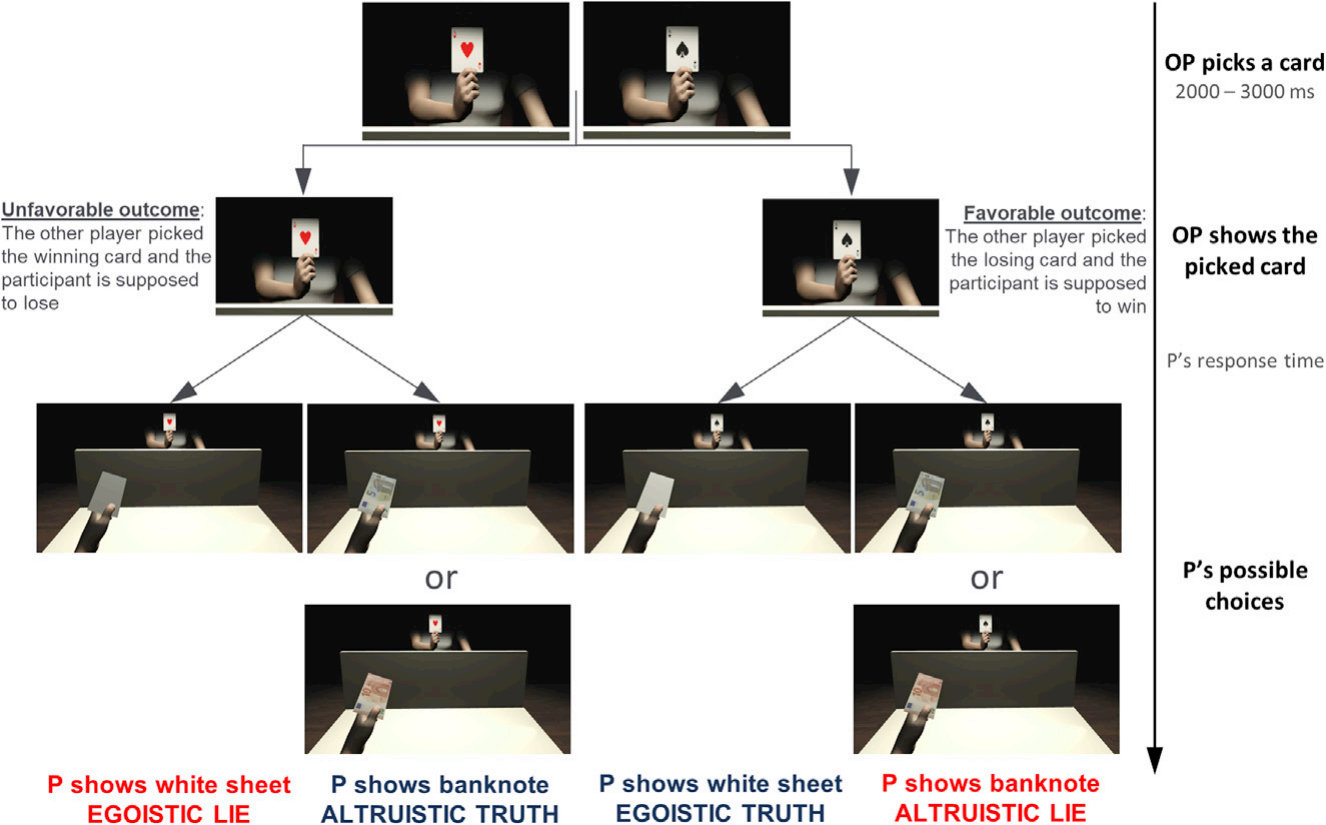
Schematic representation of the virtual reality version of the Temptation to Lie Card Game (VR-TLCG) A timeline of each trial is presented on the rightmost part of the figure. The other player (OP) picks one of two covered cards and the OP’s avatar shows the drawn card to the participant (P) within the virtual environment. If the lifted card is the ace of hearts (left side of the figure), this represents an unfavorable situation for the P. If the OP picked the ace of spades (right side of the figure), this indicates a favorable situation for the P. The P can now decide whether to lift the hand holding a virtual, white piece of paper or the one holding a virtual banknote. Both paper and banknote appeared on the left (right) hand of the P’s virtual body on half of the trials. By lifting the white piece of paper, the Ps are communicating that the OP lost (and they won). To communicate that the OP won (and they lost), the Ps can lift the virtual banknote. In 50% of trials, the virtual banknote resembled a 5€ (10€) banknote, indicating a low reward (high reward) trial.

**Figure 2:**
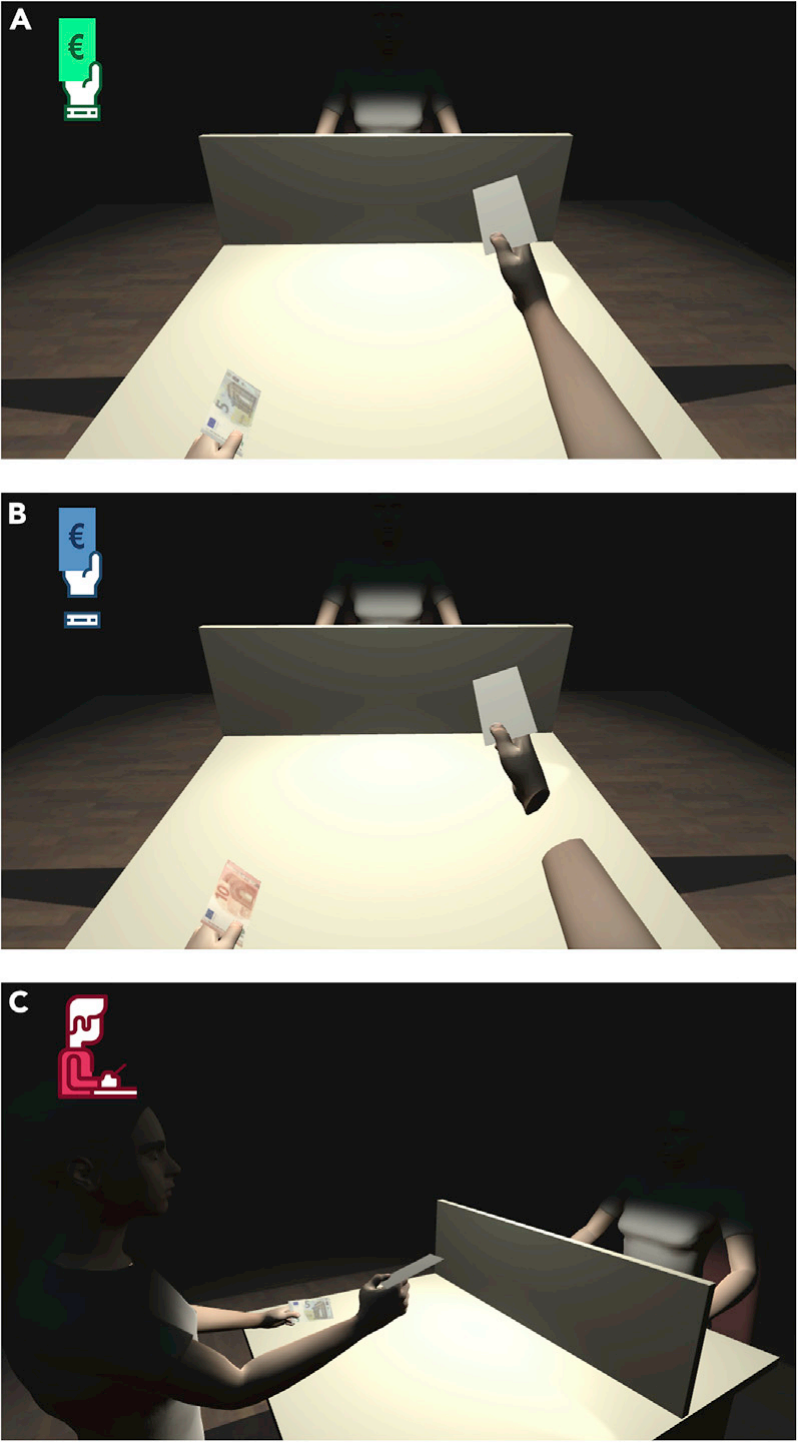
Visual representation of the experimental conditions the participants completed the VR-TLCG in (A) *1PP-Wrist* condition (congruent perspective, hands attached to the rest of the virtual body); B) *1PP-No Wrist* condition (congruent perspective, hands visually separated from the virtual avatar); C) *3PP-Wrist* condition (out-of-body perspective, hands attached). Each panel shows which icon refers to a specific experimental condition in all the following figures included in this paper.

In the present study, we aimed at exploring whether reduced SoO over a virtual body could change the participants’ tendency to lie and whether this effect is modulated by the degree of negative impact the immoral decision has on others. To achieve this aim, we created a virtual reality version of the Temptation to Lie Card Game (VR-TLCG), a task where participants interact with other individuals and can freely decide whether to engage in deception or honesty (Azevedo et al., 2017; Panasiti et al., 2011, 2014, 2016; Parisi et al., 2021; Schepisi et al., 2020). In this task, participants communicate the outcome of card picks performed by another person, knowing that their own and the other person’s compensations depend on what they decide to communicate (and not on actual picks). Crucially, in each and every trial, only the participant or the picker can obtain the money at play. Thus in *unfavorable situations* (i.e., when participants are supposed to lose), they could lie egotistically and obtain the money, or in *favorable situation* (when participants are supposed to win), they could lie altruistically and give the monetary reward to the other player (see Figure 1 for a schematic representation of the VR-TLCG). All participants were asked to perform the VR-TLCG by means of: i) an intact virtual body seen as if it were their own, that is, from a congruent point of view (first-person perspective) (*1PP-Wrist*); ii) an intact virtual body, seen from its right side (third-person perspective) (*3PP-Wrist*); and iii) a virtual body, seen from a congruent point of view (first-person perspective) but with the acting virtual hands detached from the forearm (*1PP-No Wrist*). See Figure 2 and Video S1 for a visual representation of these three conditions. Crucially, to unequivocally attribute modulations of the participants’ tendency to lie to variations in the SoO (and not in the SoA), all conditions entailed that the participants always had control over the avatar’s movements and thus had in principle the same SoA in all the conditions. To check that our manipulations successfully modulated SoO, we asked participants to rate different statements concerning their feelings of ownership, agency and location along separate Visual Analogue Scales (VAS) (all statements rated by participants are reported in Table 1), and to do so before and after completing the VR-TCLG in each experimental condition (i.e., *1PP-Wrist*, *1PP-No Wrist,* and *3PP-Wrist*). To test whether the magnitude of rewards could moderate the relation between the SoO and moral behavior, reward values could be high or low in different VR-TLCG trials.

**Table 1:** Statements assessing the participant’s level of embodiment toward the virtual bodies

	Measured component	Statements presented to participants
VAS1	Ownership	I felt as if the virtual body with banknotes in its hands was my body
VAS2	Ownership	It felt as if the virtual body with banknotes in its hands was someone else’s
VAS3	Ownership	It seemed as if I might have more than one body
VAS4	Agency	It felt like I could control the virtual body with banknotes in its hands as if it was my own body
VAS5	Agency	The movements of the virtual body with banknotes in its hands were caused by my movements
VAS6	Agency	I felt as if the movements of the virtual body with banknotes in its hands were influencing my own movements
VAS7	Agency	I felt as if the virtual body with banknotes in its hands was moving by itself
VAS8	Location	I felt as if my body was located where I saw the virtual body holding banknotes
VAS9	Location	I felt out of my body
VAS10	Location	I felt as if my real body were drifting toward the virtual body with banknotes in its hands or as if the virtual body with banknotes in its hands were drifting toward my real body

The statements were presented twice for each experimental condition (i.e., *1PP-Wrist*, *1PP-No Wrist*, and *3PP-Wrist*), namely before and after the VR-TLCG blocks. The statements appeared in randomized order. To make sure participants provided ratings concerning their avatar (and not the other player’s avatar), the statements included a reference to the “virtual body with banknotes in its hands,” when relevant. See Table S10 for the Italian version of statements.

## Results

We observed a non-normal distribution of the percentage of lies during the VR-TLCG, and ofownership, agency, and location ratings, which all constituted our dependent variables. In addition, the residual errors of separate, exploratory ANOVA models over our dependent variables also showed a significant deviation from normality (see STAR Methods section and Table S1 and Table S2 for detailed results of the Shapiro-Wilk tests of normality). Thus, data were analyzed through Friedman tests followed by post hoc, Wilcoxon signed-rank tests. We applied Bonferroni correction for multiple comparisons.

### Ownership ratings are reduced in *3PP-Wrist*

**Figure 3:**
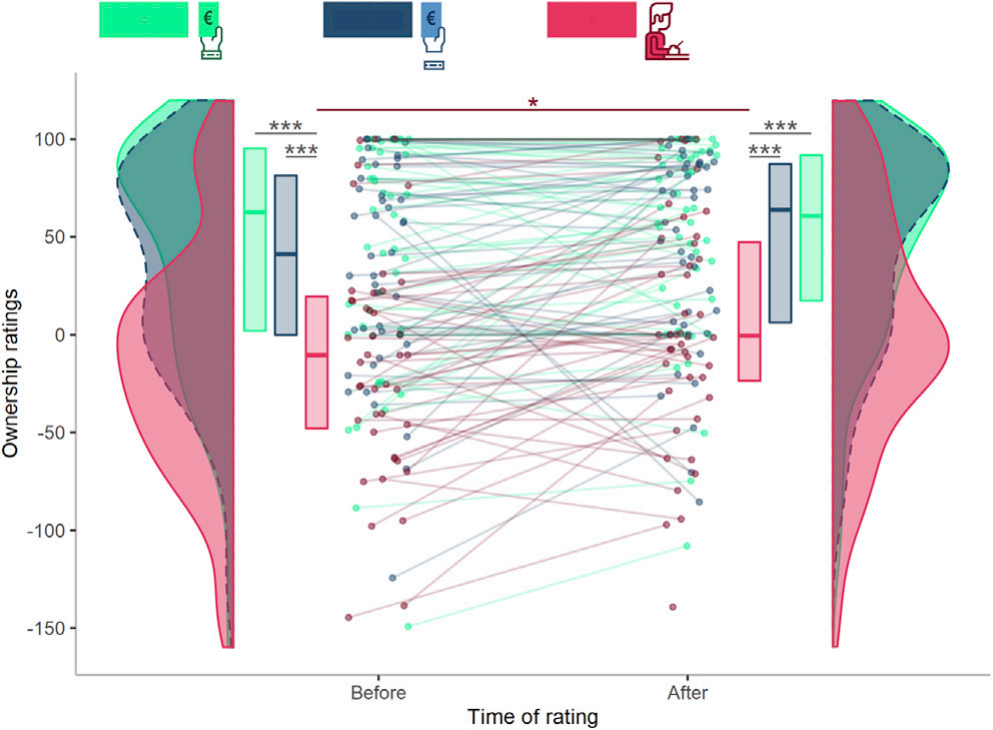
Ownership ratings are reduced in *3PP-Wrist* Ownership ratings in each experimental condition and their change over time. Violin plots represent the distribution of ownership ratings for each experimental condition. The thick horizontal lines within the boxes indicate the median, whereas the lower and upper ends of the boxes represent Quartile_1_ and Quartile_3_, respectively. Connected dots represent how the ratings of the same participant changed over time for each experimental condition. Asterisks indicate significance (∗p < 0.05, ∗∗∗p < 0.001). See Tables S3 and S4 for additional details.

To check whether the *1PP-No Wrist* and *3PP-Wrist* manipulations had successfully reduced the participants’ SoO toward the virtual bodies, we ran a Friedman test over ownership ratings. Results indicated a significant difference in the distribution of the dependent variable between the 6 combinations of factors *condition* (i.e., *1PP-Wrist*, *1PP-No Wrist,* or *3PP-Wrist*) and *time of rating* (*before* or *after* the VR-TLCG) (χ^2^ (degrees of freedom = 5) = 64.106, Kendall’s W = 0.291, 95% Confidence Intervals or CI [0.17, 0.45], p < 0.001). Post hoc comparisons revealed that independently from the time of rating, the feeling of owning the virtual body was lower in the *3PP-Wrist* condition in comparison to the *1PP-Wrist* condition (Before: Z = 4.53, *r* = 0.687, 95% CI [0.51, 0.83], p < 0.001; After: Z = 4.37, *r* = 0.642, 95% CI [0.43, 0.79], p < 0.001). Similarly, ratings associated with the *3PP-Wrist* condition were significantly lower than those reported in the *1PP-No Wrist* condition (Before: Z = 4.50, *r* = 0.689, 95% CI [0.50, 0.83], p < 0.001; After: Z = 4.18, *r* = 0.639, 95% CI [0.43, 0.79], p < 0.001). The *1PP-Wrist* and *1PP-No Wrist* conditions did not differ in terms of ownership (Before: Z = 1.06, *r* = 0.163, 95% CI [0.01, 0.45], p = 1.00; After: Z = −0.24, *r* = 0.039, 95% CI [0.01, 0.37], p = 1.00). In the *3PP-Wrist* condition, ratings increased over time (Z = −3.19, *r* = 0.489, 95% CI [0.21, 0.72], p = 0.021) (Figure 3). See Table S3 and Table S4 for additional information.

### Agency ratings are stable across condition and time

**Figure 4:**
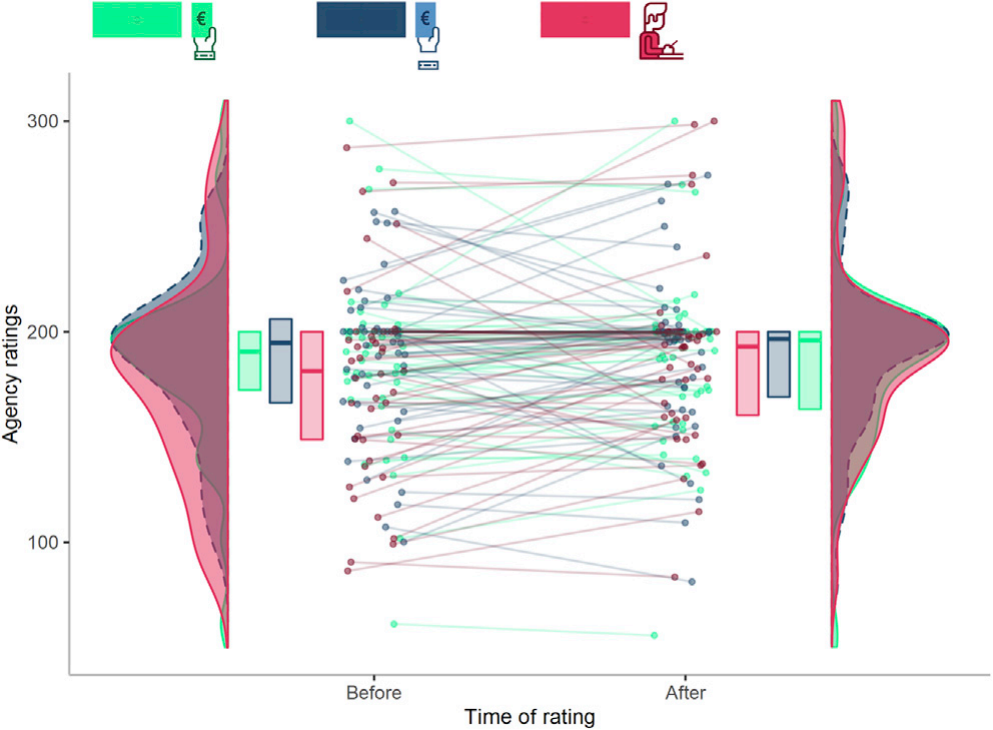
Agency ratings are stable across condition and time Agency ratings in each experimental condition and their change over time. Violin plots represent the distribution of agency ratings for each experimental condition. The thick horizontal lines within the boxes indicate the median, whereas the lower and upper ends of the boxes represent Quartile_1_ and Quartile_3_, respectively. Connected dots represent how the ratings of the same participant changed over time, for each experimental condition. See Table S5 for more details.

As expected, a Friedman test indicated that agency ratings were not different among the 6 combinations of *experimental condition* and *time of rating* (χ^2^ (5) = 8.597, W = 0.040, 95% CI [0.02, 0.13], p = 0.126) (Figure 4). See Table S5 for more details regarding the distribution of SoA ratings.

### Location ratings are reduced in *3PP-Wrist*

**Figure 5:**
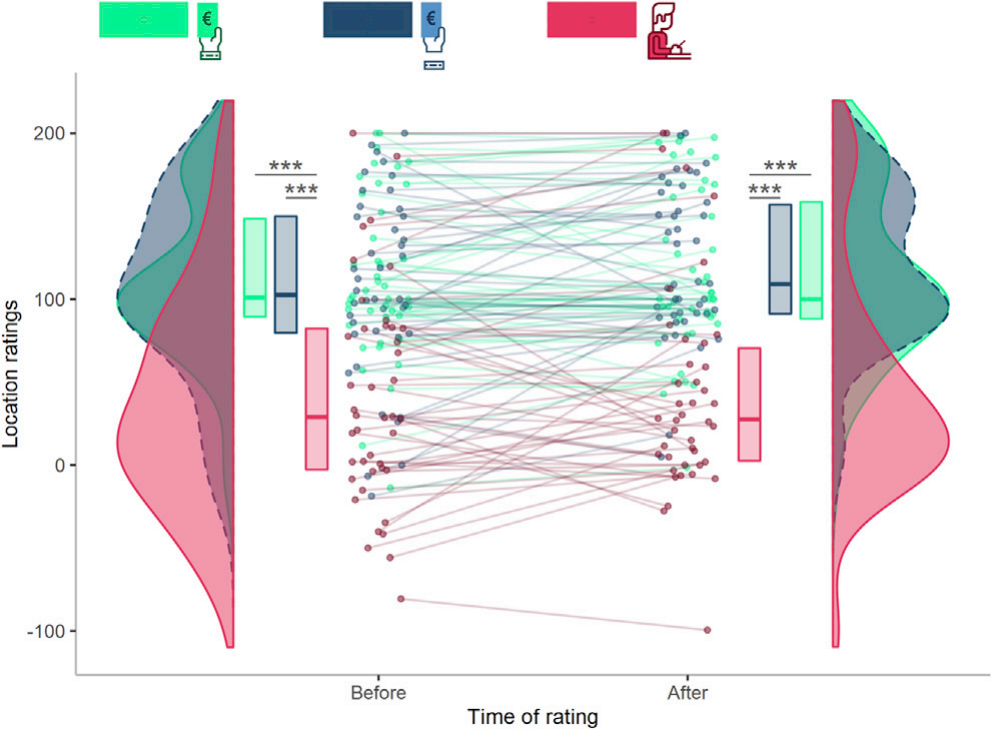
Location ratings are reduced in *3PP-Wrist* Location ratings in each experimental condition and their change over time. Violin plots represent the distribution of location ratings for each experimental condition. The thick horizontal lines within the boxes indicate the median, whereas the lower and upper ends of the boxes represent Quartile_1_ and Quartile_3_, respectively. Connected dots represent how the ratings of the same participant changed over time, for each experimental condition. The asterisks indicate significance (∗∗∗p < 0.001). See Tables S6 and S7 for further details.

To investigate whether the sense of location differed between the three experimental conditions, location scores were analyzed by means of a Friedman test. The distribution of location ratings significantly differed between the 6 combinations of *experimental condition* and *time of rating* (χ^2^ (5) = 81.637, W = 0.389, 95% CI [0.25, 0.56], p < 0.001). Wilcoxon post hoc comparisons revealed that independently from the time of rating, the *3PP-Wrist* condition had lower location ratings than the *1PP-Wrist* condition (Before: Z = 5.25, *r* = 0.814, 95% CI [0.71, 0.86], p < 0.001; After: Z = 4.95, *r* = 0.763, 95% CI [0.61, 0.86], p < 0.001). Similarly, ratings in the *3PP-Wrist* condition were lower than those associated with the *1PP-No Wrist* condition (Before: Z = 4.50, *r* = 0.694, 95% CI [0.50, 0.83], p < 0.001; After: Z = 4.82, *r* = 0.750, 95% CI [0.61, 0.85], p < 0.001). The *1PP-Wrist* and *1PP-No Wrist* conditions did not differ at either time point. Finally, in all three conditions, location ratings did not change over time (Figure 5). See Table S6 and Table S7 for further details.

### Sense of ownership and body discontinuity affect moral behavior

**Figure 6:**
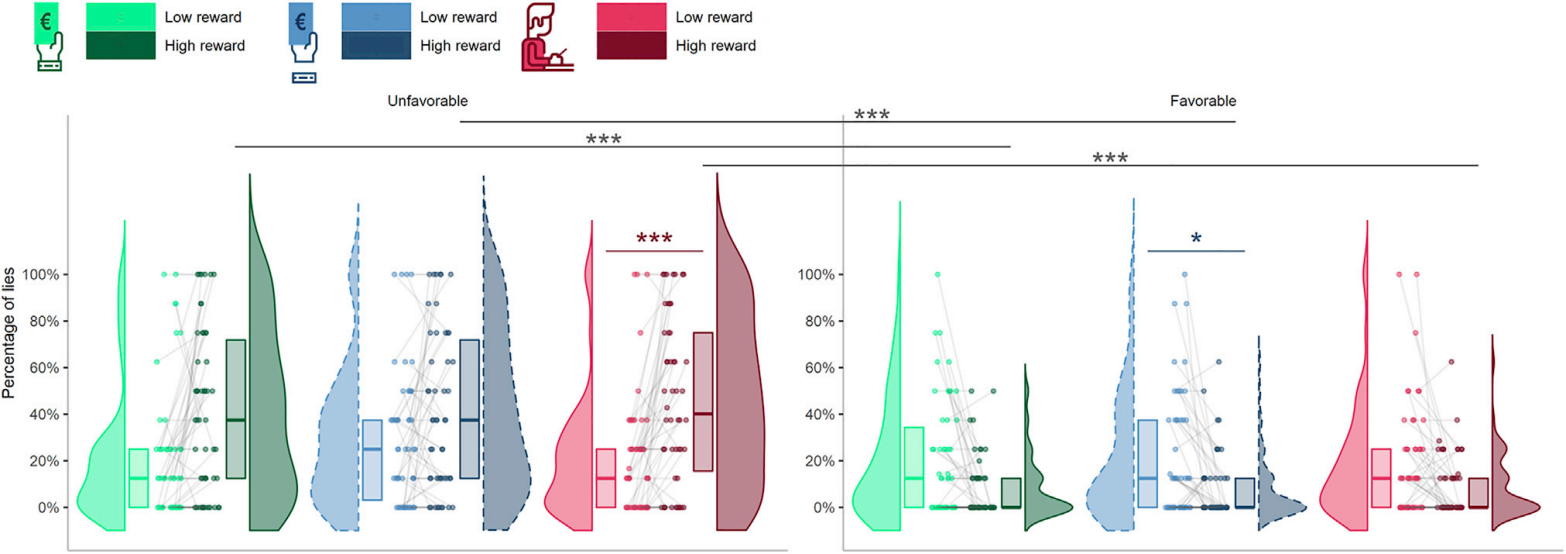
Sense of ownership and body discontinuity affect moral behavior The plot represents the percentage of lies as a function of the situation (*Favorable* or *Unfavorable* to participants), the monetary reward associated with the trial (*high* and *low*) and the experimental condition (*1PP-Wrist*, *1PP-No Wrist*, *3PP-Wrist*). Violin plots represent the distribution of lies in each experimental condition. The thick horizontal lines within the boxes indicate the median, whereas the lower and upper ends of the boxes represent Quartile_1_ and Quartile_3_, respectively. Connected dots represent how the percentage of lies communicated by the same participant changed across reward value, in each experimental condition. The asterisks indicate significance (∗p < 0.05, ∗∗∗p < 0.001). See Table S8 and Table S9 for more details.

The percentage of lies during the VR-TLCG were the dependent variable of a Friedman test assessing whether the 12 combinations of the factors *situation* (*Unfavorable* or *Favorable* to the participant), *reward value* (*low* or *high monetary reward*), and *experimental condition* (*1PP-Wrist*, *1PP-No Wrist,* and *3PP-Wrist*) differed in terms of how moral decisions were distributed. This test was significant (χ^2^ (11) = 119.739, W = 0.218, 95% CI [0.14, 0.33], p < 0.001). In trials with *high rewards*, the percentage of lies was higher in the *unfavorable situation* (i.e., egoistic lies) than in the *favorable situation* (i.e., altruistic lies). This was true for all experimental conditions (*1PP-Wrist*: Z = 4.77, *r* = 0.684, 95% CI [0.52, 0.80], p < 0.001; *1PP-No Wrist*: Z = 4.79, *r* = 0.706, 95% CI [0.55, 0.80], p < 0.001; *3PP-Wrist*: Z = 5.04, *r* = 0.720, 95% CI [0.58, 0.81], p < 0.001). In addition, only in the *3PP-Wrist* condition, the percentage of egoistic lies was higher in *high reward* trials than in *low reward* trials (Z = −4.58, *r* = 0.646, 95% CI [0.47, 0.78], p < 0.001). Finally, during the *1PP-No Wrist* condition, *high rewards* were associated with a lower percentage of altruistic lies in comparison with *low rewards* (Z = 3.63, *r* = 0.473, 95% CI [0.25, 0.67], p = 0.020) (Figure 6).

Video S1. Video showing the Motion Capture technology employed in this study and the three Experimental conditions under which participants completed the VR-TLCG, related to STAR Methods section

## Discussion

In the present study, we investigated whether changes in the SoO over a virtual avatar can bias the tendency to lie in social situations. We did so by creating an immersive virtual reality paradigm that proved adept at manipulating the SoO toward a virtual body, whereas participants were playing a card game with other individuals. The card game was a virtual version of the TLCG (Azevedo et al., 2017; Panasiti et al., 2011, 2014, 2016; Schepisi et al., 2020), a task where, by lying or telling the truth, participants not only determine their own gains but also the other players’. To clarify if rewards of different magnitudes can impact the hypothesized relation between SoO and (dis)honest behavior, each trial of the VR-TLCG was associated with a high or low monetary reward.

We found that ownership ratings were reduced when participants observed a virtual body from a third-person perspective. Although ownership ratings in the *3PP-Wrist* condition increased over time, they remained consistently lower than in the other two conditions (*1PP-Wrist*; *1PP-No Wrist*). This means that playing the game while watching one’s own avatar from a third-person perspective successfully impaired participants’ SoO toward it. This result is in line with past research, where a viewpoint located outside the body was found to diminish the SoO in comparison with a congruent, first-person perspective (Maselli and Slater, 2013; Monti et al., 2020). The same perspective-related reduction of body ownership has been reported also in conditions of preserved visuo-motor synchrony, that is, even when participants could control the movements of a virtual avatar in real time (Gorisse et al., 2017), as in the case of our study. When in the *3PP-Wrist* condition, participants also reported lower feelings of location in comparison with the other two conditions. Although evidence appears to support the notion that the SoO and the sense of location are two separate components of bodily self-consciousness, experimentally induced enhancement or reduction of the latter seem to also produce changes in the reported SoO (Guterstam et al., 2015; Serino et al., 2013). In line with this, both sense of location and of ownership remained intact in those conditions where the first-person perspective over the virtual body was maintained (i.e., *1PP-Wrist* and *1PP-No Wrist*). Contrary to our hypothesis, visual discontinuity of the upper limbs (*1PP-No Wrist*) did not modulate participants’ SoO over the avatar. Indeed, ownership ratings did not differ between this condition and the control one (i.e., *1PP-Wrist*). This result stands in contrast with those of previous studies, where observation of hands that are visually detached from a virtual body diminished the feelings of ownership (Fusco et al., 2021; Perez-Marcos et al., 2012; Tieri et al., 2015a, 2015b). In these studies, however, participants were only passively observing the visually detached hand. On the contrary, participants who took part in the present experiment had control over the movements of the avatar. Thus, visuomotor synchrony may constitute one possible explanation for the difference between past results and those presented here. In support of this hypothesis, Brugada-Ramentol et al. (2019) found that passive observation of a static, detached hand (vs. intact hand) was associated with reduced ownership, but that this effect disappeared when participants were allowed to move their real arm and its movements corresponded to those of the detached, virtual arm.

Importantly, and as we had anticipated, SoA ratings were not affected by our experimental manipulations. That is, participants always reported feeling in control of the virtual body irrespective of perspective (*i.e., 3PP-Wrist*) and of body continuity (i.e., *1PP-No Wrist*). This result is in line with evidence suggesting that the SoA is preserved in the presence of visuomotor synchrony (Caspar et al., 2016; David et al., 2016; Villa et al., 2018, 2021; Weiss et al., 2014) and even when the movements of an artificial limb are observed in an incongruent position with respect to that of one’s own limb (Kalckert and Ehrsson, 2012; Ratcliffe and Newport, 2017). The fact that ratings of ownership, and not those of agency, were reduced by external observation of a virtual body confirms that our paradigm successfully modified the SoO while leaving the SoA intact.

The results of the present study show that when high rewards were at stake, our participants engaged in a higher number of egoistic lies (i.e., keeping the highest payoff to themselves) in comparison with altruistic lies (which allocated the reward to the other player). We observed this under all three experimental conditions. Studies without modulations of the SoO show a similar increase of dishonest behaviors when individuals find themselves in unfavorable situations (Panasiti et al., 2011, 2014; Parisi et al., 2021), and when doing so entails high rewards (Gerlach et al., 2019). What is particularly interesting about our findings is that the same pattern appears to apply also when the SoO is reduced (like in the *3PP-Wrist* condition), even when the participants feel like the person in the unfavorable situation is not *fully* themselves. Crucially, despite having a sense that the body was not theirs, participants still experienced SoA and therefore a role in determining the actions of the avatar, which in turn had a real impact on their own, final retribution. Consistently, SoA is considered a fundamental pillar of the Self (Gallagher, 2000), suggesting that participants may still have (at least partially) identified with the avatar, even when their SoO was reduced. All this appears to suggest that the possibility of increasing one’s payoff by way of controlling an external, virtual body is tempting enough to overcome SoO reductions.

In addition, we found that compared to low rewards, high rewards were associated with a higher percentage of egoistic lies but only when the SoO was reduced, that is, only in the third-person perspective condition (*3PP-Wrist).* This result may highlight that similar to other embodied mechanisms, the sense of body ownership may contribute to preserving the moral self-image. As an example, research has shown that when individuals behave immorally or are reminded of past unethical behaviors, they increase their preference for cleansing products (Lee and Schwarz, 2010; Zhong and Liljenquist, 2006). This effect, known as moral cleansing, has been thought of as an attempt to reestablish a threatened concept of self through physical cleanness. Accordingly, allowing participants to use cleansing products appears to reduce their feelings of guilt (West and Zhong, 2015). In line with this, a recent study from our group showed that inducing deontological guilt (and giving no cleansing option) increases egoistic behaviors, especially in those who are high in disgust sensitivity (Parisi et al., 2021). However, in a recent review, Lee and Schwarz (2021) have proposed a new interpretation of these results, arguing that moral cleansing represents one of the many mechanisms through which people separate the self from stimuli or events, especially negative ones. In this view, it is possible that reductions of the SoO may serve the same purpose, that is, distancing from negative attributes and therefore reduce their effects (Scattolin et al., 2021a). In line with this hypothesis, Pyasik and Pia (2021) have recently shown that the physiological response to observed dishonesty was reduced when individuals did not experience SoO toward a lying, virtual body. On the contrary, in conditions where the virtual body felt more like their own, participants’ skin conductance responses were larger when the avatar lied than when a truthful response was observed. Crucially, the latter pattern of results was also found when lies and truthful responses were given by participants themselves. In other words, feeling SoO over a dishonest, virtual body originated the same physiological reactions one would observe if participants were lying themselves. On the other hand, such physiological reactions were not observed when the SoO was reduced, which goes to show that reductions of the SoO may facilitate separation from negative elements, such as immoral actions. Accordingly, diminished body ownership has been observed also in association with reductions of fear (Bourdin et al., 2017; Hofer et al., 2017) and pain sensitivity (Martini et al., 2014), as well as following (observed) motor errors (Pezzetta et al., 2018). The link we found between diminished body ownership and our participants’ dishonest behavior may be further proof in favor of a connection between bodily self-consciousness and higher-level psychological functions. Specifically, one possibility is that the reduction of ownership experienced in the *3PP-Wrist* condition may have allowed participants to distance themselves from the virtual body and consequently from the negative attributes associated with it. In other words, because it was not perceived as their own, the behaviors enacted by the virtual body were not perceived as a threat to the participants’ own moral image, thus making more dishonest behaviors – i.e., lies through which the highest reward could be obtained – less problematic. Crucially, this pattern may appear to be the opposite to that found in the literature, which instead suggests that individuals act less dishonestly in the presence of high rewards (Cohn et al., 2019; Mazar et al., 2008). However, at a closer look, our results may complement rather than contradict previous studies. Indeed, people are generally believed to aim at preserving their self-image while still allowing themselves some dishonesty (Mazar et al., 2008). To simultaneously achieve these two goals, individuals rely on different mechanisms of self-concept maintenance. On the one hand, when personal moral standards are not the primary focus of attention, individuals are less likely to compare their (immoral) conduct to their standards and to update their image of self based on this comparison. On the other, individuals may engage only in behaviors that can be more easily reinterpreted as a proof of their own morality, such as when dishonesty earns low (vs. high) rewards. We propose that a reduction of body ownership may fall into the latter mechanism and provide another convenient source of categorization malleability. Because the deceptive body is not felt as one’s own, it may become easier to reinterpret immoral behaviors as independent from one’s moral standard. This would consequently allow for greater dishonesty (in this case, for a high reward), without threatening one’s moral image. Despite our participants showing different proportions of egoistic lies in *3PP-Wrist*, their dishonest behavior did not differ across experimental conditions. The absence of significant results suggests caution when drawing conclusions on the specific effects of different degrees of SoO over (im)moral choices, an aspect that future studies should indeed address further. Interestingly, the number of egoistic lies did not differ between low and high rewards in the other two experimental conditions, that is, *1PP-Wrist* and *1PP-No Wrist*. Instead, we found that the number of altruistic lies associated with high rewards was smaller than those involving low rewards when in the presence of body discontinuity (*1PP-No Wrist*). Although reduced body ownership may explain our results concerning dishonest, egoistic behaviors in the *3PP-Wrist* condition, we cannot draw the same conclusion in the case of visual discontinuity between the virtual hands and arms. In fact, this condition was not associated with any reduction (nor increase) of the SoO in comparison to that reported in the control condition (*1PP-Wrist*). Nevertheless, it was only when appearance of the body was implausible (i.e., hands were disconnected from the rest of the body) that high rewards reduced altruistic lies. We argue that merely seeing virtual hands detached from the rest of the body may also have cued a sense of separation between the self and the negative outcome of egoistic decisions, albeit to a different extent. It is possible that, because the SoO of participants was intact, the feeling of separation originated by the disconnection of the virtual hands was enough only to justify self-serving, but not dishonest, behaviors. In fact, and in contrast to egoistic lies, a reduction of altruistic behaviors constituted the legitimate way for the participants to increase their own final payoff, as this result was achieved by providing truthful responses.

Overall, our study suggests that the SoO over the body may play a role during moral decision-making and that this relationship may be modulated by the extent of monetary rewards. The data presented here support the notion that reducing body ownership may bias decisions toward self-serving, dishonest behaviors when rewards at stake are high. In fact, people may rely on low feelings of embodiment to distance the self from the negative consequences of their actions, ultimately making dishonesty less challenging for their own moral integrity. At the same time, mere visual discontinuity may favor self-serving behavior by reducing the frequency of altruistic lies in the presence of high rewards.

Our results are especially relevant when considering that an increasing number of human activities occur online, where individuals do not interact through their bodies. In fact, interactions based on telepresence and on online applications have received further boost from measures aimed at COVID-19 pandemic containment and from a recent increase of interest and investments toward the development of metaverses, i.e., shared 3D worlds where individuals that are physically separated can interact through virtual representations. In light of this, understanding whether reductions of ownership toward a virtual object/avatar can impact our social behavior appears to be extremely relevant. Our results suggest that conditions where the SoO is reduced may facilitate a sense of separation from dishonest behaviors in online contexts. However, it may be possible to contain this tendency by inducing ownership over virtual, online characters. In situations such as multiplayer 3D games, this may be aided by a first-person perspective over the avatars that individuals use to communicate with others. At the same time, it is possible that VR interactions may benefit even further from representing users through bodies that are visually attached to the effectors, a condition that may preserve altruistic behaviors.

### Limitations of the study

The results presented here show that the SoO was reduced exclusively when participants had an out-of-body perspective over their virtual avatar (in the *3PP-Wrist* condition). Besides, in contrast with previous evidence (Fusco et al., 2021; Perez-Marcos et al., 2012; Tieri et al., 2015a, 2015b), visual discontinuity between the virtual arms and hands (*1PP-No Wrist*) did not lower the SoO of participants. However, it should be noted that the questions we employed for the assessment of body ownership always referred to “the virtual body” and not to “virtual hands.” This choice was motivated by our interest in the SoO that the participants experienced toward the full virtual body (vs. its parts). However, it could be argued that participants may indeed have felt ownership over the virtual body but could have reported a reduced SoO for the detached hand, if asked specifically about it. In light of this, future studies should consider combining questions related to the whole body with those pertaining to specific parts of the virtual and physical body to obtain a granular assessment of embodiment under different conditions.

Considering that technical issues prevented us from reliably using: i) a subset (0.69%) of the total VR-TLCG trials, and ii) the ownership, agency, and location ratings of 6, 7, and 8 participants, respectively, these data were not included in the datasets used in our analysis.

## STAR★Methods

### Key resources table

**Table:** 

REAGENT or RESOURCE	SOURCE	IDENTIFIER
**Deposited data**		

Data from 50 human participants and code for replication of analyses	This paper; Mendeley Data	Mendeley Data: https://doi.org/10.17632/2jmjp36p3t.1

**Software and algorithms**		

Unity (version 2018.3.3f1)	Unity Technologies	https://unity.com/
MakeHuman (version 1.1.1)	The MakeHuman Team (2017)	http://www.makehumancommunity.org/
Autodesk 3ds Max 2017 (version 19.0 student)	Autodesk Inc.	https://www.autodesk.it/products/3ds-max/overview?term=1-YEAR&tab=subscription
MVN Studio (version 3.5.3)	Xsens® b.v.; Roetenberg et al. (2009)	https://www.xsens.com/
PsyToolkit (version 2.5.1)	Stoet (2017); Stoet (2010)	https://www.psytoolkit.org/
R (version 4.1.3)	R Core Team (2022)	https://www.R-project.org/
RStudio (version 2022.02.0)	RStudio Team (2022)	http://www.rstudio.com/

### Resource availability

#### Lead contact

Further information and requests should be directed to and will be fulfilled by the lead contact, Marina Scattolin (marina.scattolin@iit.it).

#### Materials availability

This study did not generate new unique materials.

### Experimental model and subject details

#### Participants

The final sample included 50 participants (females = 25) whose age ranged from 19 to 30 (M = 23.1, SD = 2.565). Because we did not have any specific hypothesis concerning the possible role of biological sex in the relation between SoO and moral behavior, we did not run any specific analysis concerning this aspect. Due to the limited range of the motion capture suits size, all participants had a Body Mass Index between 18 and 25. None of the participants reported ever experiencing motion sickness in VR, having any known neurological and psychiatric condition or being taking any psychiatric drug at the time of testing; all had normal or corrected to normal vision and gave their informed consent to participation to the study and to use of their data. Also, none of the participants had played any version of the TLCG before.

Prior to recruitment, the Ethics Committee of the IRCCS Fondazione Santa Lucia (Rome) approved the methods and procedures of this study (protocol number CE/PROG.659), which are in conformity with the ethical principles of the Declaration of Helsinki. All participants were compensated for taking part in the study.

### Method details

#### Materials

An immersive virtual reality version of the Temptation to Lie Card Game (VR-TLCG) (Azevedo et al., 2017; Panasiti et al., 2011, 2014, 2016; Schepisi et al., 2020) was implemented using Unity software (Unity Technology, version 2018.3.3f1) and presented to all participants via the HTC VIVE Head Mounted Display (HMD) (https://www.vive.com/eu/product/vive/). The avatars presented in the different scenarios were created in MakeHuman (The MakeHuman Team, version 1.1.1) and modified with Autodesk 3ds Max 2017 (Autodesk Inc., version 19.0 student). For the duration of the task, participants were wearing a motion capture (MoCap) system (Xsens MVN Wired Version) which consisted of 17 sensors secured to the body by means of Velcro straps and wired to one another, each continuously measuring the linear and angular motion of the body part it was attached to. Information regarding movements of each sensor was sent to Unity via MVN Studio software (Xsens® b.v., version 3.5.3) (Roetenberg et al., 2009) and used to animate the avatar of participants so that movements temporally and spatially matched those of the person wearing the MoCap system. The participants used a footswitch (PCsensor FS2016-USB2) to rate a series of statements concerning their sense of ownership, agency and location. The final questionnaire was programmed and completed by participants using the online version of PsyToolkit (2.5.1) (Stoet, 2010, 2017).

#### Virtual temptation to lie card game (VR-TLCG)

Participants were told that they would play a card game with three other volunteers, each of whom had taken part in the study in the preceding days and had been randomly assigned to play in the “card picker” role. Pickers had been asked to repeatedly select one of two covered cards, where the ace of hearts indicated that the picker won the current round and the monetary sum associated with it (*situation unfavorable to the participant*), and the ace of spades indicated that the picker lost the current round and that the money had been won by the participants themselves (*favorable situation*). Notably, for each round only one of the players could win. The participants were also told that pickers had not been given the opportunity to look at the cards they had drawn nor were they given any feedback regarding their picks. Instead, the sequence of cards that the pickers selected had been recorded during their session and would be shown to the participants, whose task was that of communicating the outcome of each pick to the person they were playing with. This gave participants the opportunity to decide whether to confirm the outcome of the game or to deceive the picker and reverse the outcome. Crucially, and unbeknownst to participants, the sequence of cards was randomly generated by the computer, so that the ace of hearts was purportedly chosen by the picker in 50% of the trials.

The game was played within the virtual environment, where an avatar sitting across a virtual table lifted and revealed the cards that pickers had drawn. In the virtual environment, participants were represented by an avatar whose movements spatially and temporally matched their own. This was true for all parts of the participants’ body but their fingers, the motion of which was not tracked and could not be mirrored in the virtual environment. For this reason, we asked participants to position their fingers in a way that matched the avatar’s, and to do so for the entire duration of the task. Because the avatars of participants were continuously holding a white paper sheet in one hand and a banknote in the other, participants had to keep their real fingers as if doing the same. Banknotes were held in the right virtual hand of the participants’ avatar in half of the trials and in the left hand in the other half. To inform the picker that they had drawn the winning card, participants were instructed to lift the hand holding the banknote, as if showing it to the picker; to communicate a loss, participants had to raise the hand corresponding to the white sheet. When shown the ace of hearts (*unfavorable situation*, i.e., the other person picked the winning card), participants could either lift the banknote to confirm the outcome (*altruistic truth*, i.e., the picker gets the monetary reward associated to the current trial) or raise the white paper and reverse the outcome (*egoistic lie*, i.e., the participant gets the monetary reward); oppositely, in trials where the ace of spades was drawn (*favorable situation*, i.e., the other person picked the losing card), participants could show the white paper and confirm the outcome (*egoistic truth*, the participant gets the monetary reward), or lift the banknote and thus reverse the outcome (*altruistic lie*, the picker gets the monetary reward) (see Figure 1 for a visual representation of the VR-TLCG). A response was recorded as soon as a virtual hand lost contact with the virtual table.

The virtual banknote resembled a 5 euros one in 50% of trials, and a 10 euros banknote in the remaining 50%. The appearance of the virtual banknote informed participants of which trials were associated with a *low monetary reward* (5 euros resemblance) and which with a *high monetary reward* (10 euros resemblance). However, participants were informed of the fact that they and the picker would receive small monetary amounts as a result of their decisions. In fact, the appearance of the banknote did not reflect the actual payoff associated to that trial, but rather that trials with 5 euros banknotes were associated with a relatively smaller monetary reward in comparison with trials showing 10 euros banknotes.

Participants were aware that both themselves and the pickers would receive a fixed amount of money for taking part in the study, and an additional varying sum dependent on the game itself. Crucially, participants were informed that this additional payment would be calculated based on i) the reward value associated to each trial, and ii) their decisions – i.e. the outcomes they communicated to the pickers – rather than on the cards that were actually drawn. In reality, all participants received the same compensation, independently of how they behaved during the task.

To modulate the SoO associated to the virtual body, all participants were asked to complete the VR-TLCG task under three different conditions. In one case, participants observed their virtual body as if it was their real one, that is, from a first person perspective, with wrists showing and connecting the avatar’s hands to their virtual forearms (*1PP-Wrist*). In a second condition, participants looked at their avatar from a first person perspective, but the virtual wrists were not shown, hence hands appeared detached from virtual forearms (*1PP-No Wrist*). In a yet different condition, participants observed their avatar from its right side (third-person perspective) but hands were connected to the rest of the virtual body (*3PP-Wrist*) (to see all three conditions as well as the virtual environment participants were playing in, see Video S1). For each condition (i.e., *1PP-Wrist*, *1PP-No Wrist* and *3PP-Wrist*), participants completed two blocks of 16 VR-TLCG trials, resulting in 96 trials in total. The presentation order of the three experimental conditions was counterbalanced across participants. Participants were also informed that in each experimental condition they would be shown the cards drawn by a different picker.

#### Embodiment ratings in VR

To investigate the feelings of ownership, agency and location associated with their virtual body in different experimental conditions and how these might change over time, we asked participants to rate how much they agreed with 10 of the statements reported in Gonzalez-Franco and Peck (2018). All the selected statements were adapted to the characteristics of our virtual environment and are reported in Table 1 (for the Italian version presented to participants, see Table S10). These were shown six times, that is, prior to and after playing the VR-TLCG in each condition (i.e. *1PP-Wrist*, *1PP-No Wrist*, *3PP-Wrist*). The order of statements was randomized in each presentation. To ensure that participants focused exclusively on the ratings, statements were displayed in white against a dark virtual environment. Participants rated their agreement along a Visual Analogue Scale (VAS), by positioning a cursor along a horizontal line. The left end of the line indicated total disagreement with the statement and corresponded to a score of 0, the right end of the line signaled total agreement and was associated to a score of 100. When a statement was presented, the cursor appeared in the middle of the line (corresponding to a score of 50) and participants were instructed to use a foot switch to move the cursor towards the desired position. By pressing the left/right pedal, the cursor moved to the left/right. To confirm each rating, participants had to verbally inform the experimenter, who would save their response and display the following statement.

To compute combined ownership, agency and location scores, we applied the following formulas, reported by Gonzalez-Franco and Peck (2018):

Ownership=(VAS1−VAS2)−VAS3

Agency=VAS4+VAS5+VAS6−VAS7

Location=VAS8−VAS9+VAS10

Thus, we did not separately analyze the ratings in each VAS statement. Rather, following the calculation reported above, the ownership ratings of participants could range from -200 (no ownership over the virtual body) to +100 (full ownership over the virtual body). The possible range of agency ratings went from -100 (no sense of agency for the virtual body) to +300 (full sense of agency for the virtual body). Location ratings could range from -100 (no sense of being located where the avatar was located) to +200 (full sense of being located where the avatar was located).

#### Procedure

Participants were welcomed by the experimenter, who provided them with written information regarding the study. Once participants had signed the informed consent and had granted permission to use of their data, the experimenter took their body measurements. These were necessary to ensure appropriate calibration of the MoCap system. A list of measurements and their description is provided in Table S12. Then, participants were asked to wear the MoCap suit. After the MoCap hardware had been set up, and according to the system supplier’s guidelines, *N-pose* and *Hand-pose* calibration processes were completed. To avoid possible magnetic distortions, electronics and metal objects were kept at the further possible distance from sensors. For the same reason, participants sat on a wooden chair positioned in front of a plastic table for the entire duration of the study. Once seated, participants were shown how to position their fingers during the task and then asked to wear a Head Mounted Display (HMD), which allowed them to observe the virtual environment. The virtual environment consisted of a table and two chairs placed in the center of a dimly lit room, with two avatars – of identical gender and matching the participant’s one (i.e. female avatars for female participants, male avatars for male participants) – sitting at two opposite sides of the table, facing each other’s. Participants controlled one avatar, while the other avatar represented the other player.

After participants had placed their hands on the plastic table, their point of view and the position of all virtual objects were adjusted to match their tactile sensations. For example, when participants’ real hands were touching the real, plastic table, their virtual hands were simultaneously touching the virtual table. Adjustments were made under all three experimental conditions to ensure matching from all perspectives (that is, 1PP and 3PP). After being provided with task instructions, participants completed five VR-TLCG practice trials in each experimental condition. At this point, the experimenter reminded participants that they would be allowed to lift only one hand at a time and only to communicate the outcome to the picker. Once the pickers’ avatar had lowered the previous card (signaling that participants’ choice had been recorded), participants were instructed to place the hand back on the table.

The present study was conceived as a repeated measures experiment, thus all participants performed the VR-TLCG in all conditions. Prior to each condition (*i.e., 1PP-Wrist, 1PP-No Wrist and 3PP-Wrist*) participants carried out a guided observation procedure. They were asked to observe the virtual body for 30 s, following instructions that were verbally provided by the experimenter. The experimenter read the statements reported in Table S13 as soon as they appeared on the screen, at a rate of one statement every 6 s. The order of instructions was kept constant, the only exception being that half of our participants observed the left, virtual arm first and then the right, while the other half of participants observed the virtual arms in the opposite order. This guided observation procedure was devised to ensure that participants were aware of which virtual body they would control under the current condition (i.e., whether this was the avatar with or without wrists) and from what perspective they would observe it (i.e., from a first- or third-person perspective). After the guided observation procedure, participants rated the 10 statements assessing their sense of ownership, agency and location, which were also presented following the VR-TLCG trials.

At the end of the VR-TLCG, participants were asked to fill a manipulation check questionnaire aimed at assessing the extent of their engagement in the game. In accordance with previous studies (Panasiti et al., 2016; Schepisi et al., 2020), the target question was the following: “How involved did you feel in the game?”. Participants were asked to rate their involvement on a 5-point Likert scale ranging from “Not at all” (score 1) to “Very much” (score 5). Participants who rated their engagement with values below 3 were excluded from all analyses (see Table S11 for the number of occurrences). Once they had completed the manipulation check questionnaire, the MoCap suit was taken off of participants.

At the end of data collection, all participants were debriefed on the real nature of the study and they were informed of the fact that they had actually played against the computer. After this explanation, participants were given the opportunity to confirm or refuse their permission to use the data collected from them. All participants granted permission following the debriefing.

### Quantification and statistical analysis

Data were handled and analyzed using R software (version 4.1.3) (R Core Team, 2022) via RStudio (version 2022.02.0) (RStudio Team, 2022). Sixty individuals were recruited for the study that was conducted between July 2019 and January 2020 (thus prior to the COVID-19 outbreak). 10 participants did not meet pre-defined exclusion criteria and were excluded from the analysis (see Table S11). The final sample size (N = 50) is consistent with the results of an *a priori* power analysis for repeated measures ANOVA using G∗Power software (version 3.1.9.7). This *a priori* analysis highlighted that 36 participants were needed for a small effect size (0.2), with other parameters set as follows: α = 0.05, and power = 0.8. Because we couldn’t base our sample size estimation on any former investigation of the link between SoO and moral behavior, the effect size included in this power analysis relied on the average effect reported in social psychology studies (Richard et al., 2003). Although all 50 participants included in the final sample were presented with 96 card picks during the VR-TLCG, 33 out of 4800 trials (0.69% of the total) had to be removed from the dataset following technical issues concerning the position of participants’ hands in the virtual environment. Therefore, all task-related analyses were based on the remaining trials (N = 4767), with each participant responding to 95.34 trials on average. A decision to communicate the real outcome of the pick was coded as 0, while lies were coded as 1. We then computed each participant’s percentage of lies in all 12 combinations of the independent variables, that is, situation (*Unfavorable* or *Favorable* to the participant), reward value (*low monetary reward* or *high monetary reward*) and experimental condition (*1PP-Wrist*, *1PP-No Wrist* and *3PP-Wrist*). As to ownership, agency and location ratings, these were calculated for each participant and each of the 6 combinations of experimental condition (*1PP-Wrist*, *1PP-No Wrist* and *3PP-Wrist*) and time of rating (*before* or *after* completing the VR-TLCG in each condition). Due to some technical issues with the functioning of the foot pedals, the ownership, agency and location ratings datasets included the responses of 44, 43 and 42 participants, respectively. We used function *stat.desc* of the *pastecs* package (version 1.3.21) to compute a Shapiro-Wilk test of normality for all dependent variables and to check that their skewness and kurtosis fell within the −-1.96 – 1.96 range (Field et al., 2012). The lying percentage was non-normally distributed in all 12 conditions of the VR-TLCG, as were ownership, agency and location ratings in 2 out of the 6 possible combinations of independent variables, representing 33% of cases (see Table S1 and Table S2 for detailed results of these analyses). Prior to performing the analyses reported in the Results section, we checked whether the residual errors of four, separate, repeated measures ANOVA models were normally distributed, using function *shapiro.test* of the *stats* package (version 4.2.1). The percentage of lies and the ratings of ownership, agency or location ratings were the dependent variable of each of these exploratory ANOVAs. Results showed that the four distributions of residual errors significantly deviated from normality, with all *W* ≥ 0.926 and p ≤ 0.002. Considering this, we applied a nonparametric statistical approach to all analyses using the package *rstatix* (version 0.7.0). Specifically, we used functions *friedman_test* and *friedman_effsize* to compute Friedman tests and Kendall’s W coefficients, respectively. A significant result was followed by post-hoc, Wilcoxon signed-rank tests performed through the *wilcox_test* function. The effect size *r* was calculated by means of *wilcox_effsize*. A Bonferroni correction for multiple comparisons was applied.

Details about descriptive (i.e. median, quartiles and confidence intervals) and inferential statistics (i.e. Friedman test, Wilcoxon signed-rank test and p values) can be found in the Results section, Figures 3, 4, 5, and 6, and in the Supplemental information. Alpha was set at 0.05.

## Data Availability

• The full dataset has been deposited at Mendeley Data and is publicly available as of the date of publication. DOIs are listed in the key resources table. • The code for replication of all analysis has been deposited at Mendeley Data and is publicly available as of the date of publication. DOIs are listed in the key resources table. • Any additional information required to reanalyze the data reported in this paper is available from the lead contact upon request.

## Footnotes

Supplemental information can be found online at https://doi.org/10.1016/j.isci.2022.104320.
