# Supplementary material for: Reduced ownership over a virtual body modulates dishonesty
Source: iScience. 2022 Apr 27;25(5):104320. doi: 10.1016/j.isci.2022.104320 (PMC9118670; doi:10.1016/j.isci.2022.104320)
Supplement: Document S1. Tables S1–S13 [file mmc1.pdf]

iScience, Volume *25*

Supplemental information

Reduced ownership over a virtual body modulates dishonesty

Marina Scattolin, Maria Serena Panasiti, Riccardo Villa, and Salvatore Maria Aglioti

Table S1. Results of the Shapiro-Wilk test of normality over the percentage of lies in each condition of the VR-TLCG, related to sections *Results* and *STAR Methods – Quantification and statistical analysis*. W is the Shapiro-Wilk test statistics, with significance reported in the column labelled "*p*-value". Columns "$z_{Skewness}$" and "$z_{Kurtosis}$" report the z-values for skewness and kurtosis, respectively. Asterisks indicate which values exceed the -1.96 – 1.96 range, signaling a deviation from normality.

**Experimental condition**	**Situation**	**Reward value**	**W**	***p*-value**	$z_{Skewness}$	$z_{Kurtosis}$
1PP-Wrist	*Unfavorable*	*Low*	0.742	$p < .001$	4.56 *	2.04 *
		High	0.889	$p < .001$	1.10	-1.86
	Favorable	*Low*	0.797	$p < .001$	3.54 *	0.95
		High	0.693	$p < .001$	4.77 *	2.87 *
1PP-No Wrist	*Unfavorable*	*Low*	0.859	$p < .001$	3.01 *	0.65
		High	0.894	$p < .001$	1.25	-1.81
	Favorable	*Low*	0.789	$p < .001$	3.53 *	0.85
		High	0.646	$p < .001$	5.90 *	5.60 *
3PP-Wrist	*Unfavorable*	*Low*	0.762	$p < .001$	4.96 *	3.86 *
		High	0.906	$p < .001$	0.47	-2.00 *
	Favorable	*Low*	0.771	$p < .001$	4.95 *	4.04 *
		High	0.712	$p < .001$	4.94 *	4.93 *

Table S2. Results of the Shapiro-Wilk test of normality over Ownership, Agency and Location ratings in each experimental condition and time of rating, related to sections *Results* and *STAR Methods – Quantification and statistical analysis*. W is the Shapiro-Wilk test statistics, with significance reported in the column labelled “p-value”. Columns “$z_{Skewness}$” and “$z_{Kurtosis}$” report the z-values for skewness and kurtosis, respectively. Asterisks indicate which values exceed the -1.96 – 1.96 range, signaling a deviation from normality.

Experimental condition	Time of rating	W	*p*-value	$z_{Skewness}$	$z_{Kurtosis}$
**Ownership ratings**					
1PP-Wrist	*Before*	0.872	*p* < .001	-2.77 *	0.63
	After	0.874	*p* < .001	-3.10 *	1.05
1PP-No Wrist	*Before*	0.929	*p* = .009	-1.91	-0.09
	After	0.890	*p* < .001	-2.22 *	-0.59
3PP-Wrist	*Before*	0.971	*p* = .339	-0.32	-0.26
	After	0.971	*p* = .333	-0.46	-0.65
**Agency ratings**					
1PP-Wrist	*Before*	0.909	*p* = .002	-0.23	2.96 *
	After	0.909	*p* = .002	-0.29	3.01 *
1PP-No Wrist	*Before*	0.961	*p* = .155	-0.96	-0.27
	After	0.940	*p* = .027	-0.67	0.83
3PP-Wrist	*Before*	0.965	*p* = .212	0.46	-0.27
	After	0.911	*p* = .003	1.48	1.57
**Location ratings**					
1PP-Wrist	*Before*	0.954	*p* = .092	-0.15	-0.29
	After	0.943	*p* = .038	0.39	-0.99
1PP-No Wrist	*Before*	0.968	*p* = .272	-1.09	-0.68
	After	0.947	*p* = .050	-0.66	-0.52
3PP-Wrist	*Before*	0.971	*p* = .351	1.38	-0.37
	After	0.889	*p* = .001	2.45 *	0.74

Table S3. Medians and Quartiles of ownership ratings in each of the 6 combinations of factors condition and time of rating, related to section *Results* and to Figure 3. The table also reports **a**dditional information regarding the post-hoc comparisons of ownership ratings across time, for each experimental condition**.** Mdn is the median and quartiles are reported in columns *$Quartile_1$* and *$Quartile_3$*. Z indicates the z test statistics for Wilcoxon signed-rank tests while *r* is the associated effect size with its 95% lower and upper Confidence Intervals (or CI). Significance is reported in the column labelled "p-value".

**Experimental condition**	**Time of rating**						**Z**	***r*** **[95% CI]**	***p-value***
	**Before**			**vs** **After**					
	Mdn	*$Quartile_1$*	*$Quartile_3$*	Mdn	*$Quartile_1$*	*$Quartile_3$*			
1PP-Wrist	62.67	1.95	95.31	60.73	17.42	91.82	-1.09	0.144 [0.01, 0.46]	*p* = 1.00
1PP-No Wrist	41.22	-0.07	81.46	63.86	6.31	87.32	-2.09	0.312 [0.04, 0.56]	*p* = .552
3PP-Wrist	-12.27	-53.02	18.59	-0.52	-23.55	47.35	-3.19	0.489 [0.21, 0.72]	*p* = .021

Table S4. Post-hoc comparisons of ownership ratings across experimental conditions, at different times of rating, related to section *Results* and to Figure 3. Z indicates the z test statistics for Wilcoxon signed-rank tests while *r* is the associated effect size with its 95% lower and upper Confidence Intervals (or CI). Significance is reported in the column labelled “p-value”.

**Time of rating**	**Comparison between Experimental conditions**		**Z**	***r* [95% CI]**	***p-value***
**Before**	*1PP-Wrist*	*1PP-No Wrist*	1.06	0.163 [0.01, 0.45]	$p = 1.00$
	1PP-Wrist	*3PP-Wrist*	4.53	0.687 [0.51, 0.83]	$p < .001$
	1PP-No Wrist	*3PP-Wrist*	4.50	0.689 [0.50, 0.83]	$p < .001$
**After**	*1PP-Wrist*	*1PP-No Wrist*	-0.24	0.039 [0.01, 0.37]	$p = 1.00$
	1PP-Wrist	*3PP-Wrist*	4.37	0.642 [0.43, 0.78]	$p < .001$
	1PP-No Wrist	*3PP-Wrist*	4.18	0.639 [0.43, 0.79]	$p < .001$

Table S5. Medians and quartiles of agency ratings in each of the *6* combinations of factors condition *and time of rating*, related to section *Results* and to Figure 4. Mdn is the median and quartiles are reported in columns $Quartile_1$ and $Quartile_3$.

**Experimental condition**	**Time of rating**					
	**Before**			**After**		
	Mdn	$Quartile_1$	$Quartile_3$	Mdn	$Quartile_1$	$Quartile_3$
1PP-Wrist	190.52	172.28	200.00	195.89	163.21	200.00
1PP-No Wrist	194.70	166.20	206.00	196.53	169.01	200.00
3PP-Wrist	181.32	148.85	200.00	192.93	160.37	200.00

Table S6. Medians and Quartiles of location ratings in each of the 6 combinations of factors condition and time of rating, related to section *Results* and to Figure 5**.** The table also reports **a**dditional information regarding the post-hoc comparisons of location ratings across time, for each experimental condition**.** Mdn is the median and quartiles are reported in columns $Quartile_1$ and $Quartile_3$. Z indicates the z test statistics for Wilcoxon signed-rank tests while *r* is the associated effect size with its 95% lower and upper Confidence Intervals (or CI). Significance is reported in the column labelled "p".

**Experimental condition**	**Time of rating**							**Z**	***r* [95% CI]**	***p***
	**Before**			**vs**	**After**					
	Mdn	$Quartile_1$	$Quartile_3$		Mdn	$Quartile_1$	$Quartile_3$			
1PP-Wrist	100.96	89.48	148.44		100.00	88.13	158.60	-0.538	0.081 [0.007, 0.38]	1.00
1PP-No Wrist	102.56	79.70	149.91		109.11	91.25	156.97	-2.250	0.343 [0.04, 0.58]	1.00
3PP-Wrist	28.93	-2.68	82.33		27.59	2.65	70.39	-0.758	0.120 [0.008, 0.41]	1.00

Table S7. Post-hoc comparisons of location ratings across experimental conditions, at different times of rating, related to section *Results* and to Figure 5. Z indicates the z test statistics for Wilcoxon signed-rank tests while *r* is the associated effect size with its 95% lower and upper Confidence Intervals (or CI). Significance is reported in the column labelled “p-value”.

**Time of rating**	**Comparison between Experimental conditions**		**Z**	***r*** **[95% CI]**	***p-value***
**Before**	*1PP-Wrist*	*1PP-No Wrist*	0.01	0.001 [0.003, 0.35]	$p = 1.00$
	1PP-Wrist	*3PP-Wrist*	5.25	0.814 [0.71, 0.86]	$p < .001$
	1PP-No Wrist	*3PP-Wrist*	4.50	0.694 [0.50, 0.83]	$p < .001$
**After**	*1PP-Wrist*	*1PP-No Wrist*	-1.32	0.208 [0.01, 0.49]	$p = 1.00$
	1PP-Wrist	*3PP-Wrist*	4.95	0.763 [0.61, 0.86]	$p < .001$
	1PP-No Wrist	*3PP-Wrist*	4.82	0.750 [0.61, 0.85]	$p < .001$

Table S8. Medians and Quartiles of the percentage of lies in each of the 12 combinations of factors condition, reward value and situation, related to the *Results* section and Figure 6**.** The table also reports **a**dditional information regarding the post-hoc comparisons of the percentage of lies across situation (favorable or unfavorable to the participant), for each experimental condition and reward value. Mdn is the median and quartiles are reported in columns $Quartile_1$ and $Quartile_3$. Z indicates the z test statistics for Wilcoxon signed-rank tests while *r* is the associated effect size with its 95% lower and upper Confidence Intervals (or CI). Significance is reported in the column labelled “p-value”.

**Experimental condition**	**Reward Value**	**Situation**							**Z**	***r* [95% CI]**	**p-value**
		**Favorable**			**vs**	**Unfavorable**					
		**Mdn**	**$Quartile_1$**	**$Quartile_3$**		**Mdn**	**$Quartile_1$**	**$Quartile_3$**			
1PP-Wrist	Low	12.50%	0.00%	34.38%		12.50%	0.00%	25.00%	- 0.12	0.045 [0.004, 0.33]	$p = 1.00$
	High	0.00%	0.00%	12.50%		37.50%	12.50%	71.88%	4.77	0.684 [0.52, 0.80]	$p < .001$
1PP-NoWrist	Low	12.50%	0.00%	37.50%		25.00%	3.13%	37.50%	1.64	0.234 [0.02, 0.49]	$p = 1.00$
	High	0.00%	0.00%	12.50%		37.50%	12.50%	71.88%	4.79	0.706 [0.55, 0.80]	$p < .001$
3PP-Wrist	Low	12.50%	0.00%	25.00%		12.50%	0.00%	25.00%	0.57	0.042 [0.005, 0.33]	$p = 1.00$
	High	0.00%	0.00%	12.50%		40.18%	15.62%	75.00%	5.04	0.720 [0.58, 0.81]	$p < .001$

Table S9. Post-hoc comparison of lies across reward values, within each experimental condition and in each situation, related to section *Results* and to Figure 6. Z indicates the z test statistics for Wilcoxon signed-rank tests while *r* is the associated effect size with its 95% lower and upper Confidence Intervals (or CI). Significance is reported in the column labelled “p-value”.

Experimental condition	Situation	Comparison	Z	*r* [95% CI]	p-value
1PP-Wrist	Favorable	Low vs High reward	3.15	0.416 [0.17, 0.60]	p = .112
	Unfavorable	Low vs High reward	-3.36	0.522 [0.31, 0.71]	p = .053
1PP-No Wrist	Favorable	Low vs High reward	3.63	0.473 [0.25, 0.67]	p = .020
	Unfavorable	Low vs High reward	-2.28	0.223 [0.02, 0.47]	p = 1.00
3PP-Wrist	Favorable	Low vs High reward	2.90	0.406 [0.18, 0.63]	p = .256
	Unfavorable	Low vs High reward	-4.58	0.646 [0.47, 0.78]	p < .001

Table S10. Italian version of the statements assessing the participant's level of embodiment towards the virtual bodies, related to Table 1. The statements were presented twice for each experimental condition (i.e., *1PP-Wrist*, *1PP-No Wrist*, *3PP-Wrist*). The statements appeared in randomized order, after each guided observation procedure and after completing two VR-TLCG blocks.

	**Measured component**	**Statements presented to participants (Italian version)**
VAS1	Ownership	Ho avuto l'impressione che il corpo virtuale con le banconote in mano fosse il mio
VAS2	Ownership	Ho avuto l'impressione che il corpo virtuale con le banconote in mano fosse di qualcun altro
VAS3	Ownership	Ho avuto l'impressione di avere più di un corpo
VAS4	Agency	Ho avuto l'impressione di poter controllare il corpo virtuale con le banconote in mano come se fosse il mio corpo
VAS5	Agency	I movimenti del corpo virtuale con le banconote in mano erano determinati dai miei movimenti
VAS6	Agency	Ho avuto l'impressione che i movimenti del corpo virtuale con le banconote in mano influenzassero i miei movimenti
VAS7	Agency	Ho avuto l'impressione che il corpo virtuale con le banconote in mano si muovesse da solo
VAS8	Location	Ho avuto l'impressione che il mio corpo si trovasse dove vedevo il corpo virtuale con le banconote in mano
VAS9	Location	Ho avuto l'impressione di essere fuori dal mio corpo
VAS10	Location	Ho avuto l'impressione che il mio corpo reale si avvicinasse a quello con le banconote in mano o che il corpo con le banconote in mano si avvicinasse al mio corpo reale

Table S11. Reasons for exclusion of participants, related to *STAR Methods – Quantification and statistical analysis*. 'No. excluded' indicates the corresponding number of occurrences; '% excluded' indicates the percentage of participants excluded for each reason.

Reason for exclusion	No. excluded	% excluded
Did not complete the study	1	1.67
Knew the task (and cover story) beforehand	1	1.67
Did not believe the other players were real	1	1.67
Not engaged in the task	7	11.67
**Total**	**10**	**16.67**

Table S12. Description of all body measurements taken during the study, related to *STAR Methods – Procedure*. Measures were entered in MVN Studio software to ensure appropriate calibration of the Motion Capture system.

**Measured component**	**Description**
Body Height	From floor to top of the participants' head
Foot Size	Full shoe length (from back of the heel to front of the toe)
Arm Span	From one middle finger tip to the other, with arms spread out
Ankle Height	From floor to center of the ankle
Hip Height	From floor to medium trochanter
Hip Width	Distance between the two anterior superior iliac spines
Knee Height	From floor to lateral epicondile
Shoulder Width	Distance between left and right acromions
Shoe Sole Height	Shoe sole thickness
Eyes Height	From floor to eyes while seated on the wooden chair (needed for the point of view in the third person perspective condition – *3PP-Wrist*)

Table S13. Statements read by the experimenter during the guided observation procedure, related to section *STAR Methods – Procedure*. The order of statements remained the same, the first of which appeared at the beginning of the 30 seconds observation, while all other statements were presented 6 seconds after the preceding one. Half of our participants were asked to first observe the left arm and then the right one; for the remaining half of participants, the procedure started with observation of the right arm (followed by the left one).

**English version**	**Italian version**
Look at the still left/right arm	Osserva il braccio sinistro/destro da fermo
Lift the left/right arm and observe it	Alza il braccio sinistro/destro e osservalo
Lower the left/right arm and observe the still right/left arm	Abbassa il braccio sinistro/destro e osserva il braccio destro/sinistro da fermo
Lift the right/left arm and observe it	Alza il braccio destro/sinistro e osservalo
Lower the right/left arm	Abbassa il braccio destro/sinistro
